# Case Report of Post-Operative Uvular Necrosis Following Intubation

**DOI:** 10.21980/J8065J

**Published:** 2025-07-31

**Authors:** Laryssa Patti, Jal Trivedi, Mary Rometti

**Affiliations:** * Rutgers Health Robert Wood Johnson Medical School, Department of Emergency Medicine, New Brunswick, NJ; ^ University of California, San Diego, Department of Anesthesia, San Diego, CA; † Rutgers Health Community Medical Center, Department of Emergency Medicine, Toms River, NJ

## Abstract

Uvular necrosis is an uncommon but startling complication of manipulation of the posterior oropharynx, either via instrumentation or direct suctioning. Here, we present a patient who developed uvular necrosis after an urgent intubation. Although the uvular necrosis self-resolved via the auto-amputation of the uvula, the change to the uvula was emotionally distressing to the patient. As symptoms typically develop one to two days after the initial injury to the uvula, this diagnosis may be seen in the emergency department, rather than in the inpatient hospital setting.

**Topics:**

ENT, uvular disorders, complications of intubation, uvular disease.

**Figure:**
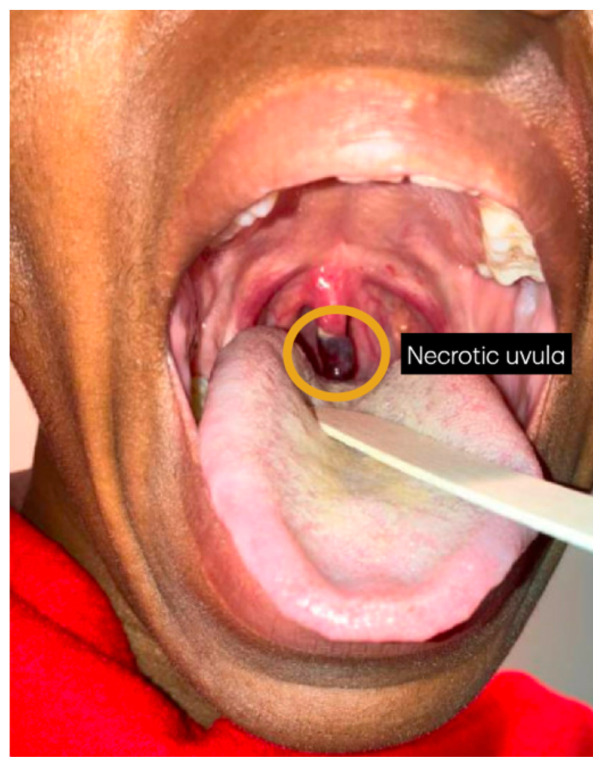


## Brief introduction

Post-intubation uvular necrosis is a rare complication with an incidence rate reported at only about 0.03%.^1^,^2^ Though usually not life-threatening, uvular necrosis can be distressing to the patient.^3^,^4^ Given the potential for complications such as edema, necrosis, and airway obstruction,^5^ uvular necrosis is an important diagnosis for emergency clinicians to recognize.

Etiology is hypothesized to be secondary to direct trauma or impingement of the vascular supply to the uvula by instrumentation or suction.^1^,^3^–^8^ Case reports describe instances of uvular necrosis following endotracheal intubation, upper gastrointestinal endoscopy, transesophageal echocardiogram, and deep oropharyngeal suctioning.^3^,^5^,^6^

Symptoms typically begin within 48 hours of instrumentation, and include dysphagia, odynophagia, globus sensation, difficulty breathing, and sore throat.^1^,^3^,^6^ The enlarged uvula may cause frequent gagging.^3^ Diagnosis is made on physical examination, where the uvula appears elongated, edematous, blackened, and may have necrotic discharge.^1^,^3^,^6^

Here, we describe the case of a patient who presented with uvular necrosis following endotracheal intubation for urgent surgical repair of an ankle fracture. Written consent for use of this photograph for educational purposes was obtained from the patient consistent with hospital policy.

## Presenting concerns and clinical findings*:*

The patient was a 35-year-old female who presented to the emergency department for swelling to the uvula, dysphagia, globus sensation, frequent gagging, and sore throat for two days. She noted that the distal portion of her uvula was discolored, prompting her presentation to the emergency department. The patient denied fevers, chills, nausea, vomiting, neck pain, or rhinorrhea.

Three days before her emergency department presentation, the patient had undergone urgent intubation in the operating suite for surgical repair of a traumatic ankle fracture. Pre-operative intubation was performed without difficulty on the first attempt via direct laryngoscopy with a grade I view of the glottis with a Macintosh size 3 blade and a 7.0 millimeter (mm) endotracheal tube. The patient was extubated at the end of her procedure and returned to baseline cardiovascular and respiratory status. The remainder of her hospitalization was uncomplicated, and she was discharged on post operative day two.

## Significant findings

On her emergency department presentation, the patient’s vital signs were within normal limits, except for a mildly elevated blood pressure, 141/98 millimeters of mercury (mmHg). On exam, she was awake, in no respiratory distress, without stridor or drooling. The distal portion of her uvula was necrotic with a clear demarcation approximately halfway up the uvula. She had no trauma to the anterior oropharyngeal structures, tonsils, or adenoids. There were no lesions to the hard or soft palate. She had no carotid bruits or thrills, and no tenderness over the anterior portion of the neck.

Blood work was performed. Complete blood count and basic metabolic panel were within normal limits, except for a hemoglobin 9.4 g/dL that was similar to the patient’s post-operative hemoglobin.

## Patient course

The patient was treated with one dose of dexamethasone intravenously (IV) in the emergency department and observed for 12 hours without change in respiratory status or worsening of uvular necrosis. The patient was seen by otolaryngology in the emergency department, who recommended discharge with dexamethasone 6 mg twice daily by mouth for three days and omeprazole 40 mg by mouth daily. The patient followed up with an otolaryngologist 10 days later. Her symptoms had improved, although she was intermittently having globus sensations. On physical examination, the uvula was noted to be thin and elongated, with a small area of eschar at the distal tip. The otherwise necrotic portions of the uvula were assumed to have auto-amputated. Uvulectomy was offered, and the patient declined.

## Discussion

Postoperative uvular necrosis may result secondary to trauma following insertion of instruments or direct suctioning and can occur across a variety of instrumentation methods including endotracheal intubation, endoscopy, or laryngeal mask airway use.^1^,^3^,^5^,^6^ Uvular necrosis appears to be more common in males and less common in pediatric patients, possibly due to differences in anatomy.^3^ The risk of uvular necrosis may be reduced by keeping the endotracheal tube and other equipment lateral to the midline, decreasing the use of blind suctioning, and using a lower suction strength.^1^–^3^,^6^

Most cases of uvular necrosis resolve with conservative treatment, usually within 14 days of the initial diagnosis, although this may be confounded by the typical two week follow-up period for operative cases.^1^,^3^ The necrotic uvula is expected to slough off spontaneously and without complication.^3^,^5^ However, case reports exist in reconstructive surgery literature in which patients developed uvular necrosis Patti L, et al. Case Report of Post-Operative Uvular Necrosis Following Intubation. JETem 2025. that became complicated by soft palate necrosis following prolonged intubation in the intensive care unit.^9^

Multiple sources suggest conservative treatment given the expected course.^3^–^6^,^9^ Corticosteroids are frequently recommended given their low risk profile and the potential for edema reduction^3^,^5^ Although there is theoretical concern for uvular super-infection, there are few documented cases of necrosis actually leading to infection, and therefore, the utility of prophylactic antibiotics is questionable.^3^ Other potential treatments include antihistamines, topical epinephrine, or observation.^5^ The patient’s presentation and severity of symptoms will aid in guiding the treatment plan and ultimate disposition.

Predictive features of high-risk patients are typically derived from case reports and retrospective series. In a retrospective review of ten cases of uvular trauma at one institution, patients with uvular trauma were predominantly male and undergoing endotracheal intubation for urologic procedures.^5^ Obesity was not associated with increased risk of uvular trauma.^5^ In Bright’s review of twenty cases from webAIRS, the voluntary web-based anesthesia incident-reporting system for Australia and New Zealand, it was noted that affected patients were also primarily male, but this time undergoing a variety of surgical procedures, and airways were obtained with both endotracheal tubes and laryngeal mask airways.^6^ All patients recovered without significant morbidity or mortality.^6^ While there are no consistent risk factors to predict this complication and no clear, definitive recommendations for the management of this condition, uvular necrosis following manipulation of the airway is a possible finding that emergency physicians should be aware of in order to appropriately counsel and treat patients. Clinicians manipulating the posterior oropharynx should consider positioning of the uvula after instrumentation to ensure adequate vascular supply.

## Supplementary Information





## References

[1] De Freitas MA Santiago Caobi L Guevara Tirado OA Uvular necrosis: Day-to-day progression of a rare postoperative complication Cureus 2023 15 9 e45132 10.7759/cureus.45132 37705569 PMC10497323

[2] Zameer S Inam SM Faisal M Iatrogenic uvular injury after endotracheal intubation: recommendations for clinical practice Ain-Shams J of Anesthesiol 2023 15 1 10.1186/s42077-023-00351-5

[3] Reid JW Samy A Jeremic G Postoperative uvular necrosis: A case series and literature review Laryngoscope 2020 130 4 880 885 10.1002/lary.28096 31145486

[4] Evans DP Lo BM Uvular necrosis after orotracheal intubation Am J Emerg Med 2009 27 5 631e3 4 10.1016/j.ajem.2008.09.004 19497485

[5] Pamnani A Faggiani SL Hood M Uvular injury during the perioperative period in patients undergoing general anesthesia Laryngoscope 2014 124 1 196 200 10.1002/lary.23774 24150972

[6] Bright MR Concha Blamey SI Beckmann LA Culwick MD Iatrogenic uvular injury related to airway instrumentation: A report of 13 cases from the webAIRS database and a review of uvular necrosis following inadvertent uvular injury Anaesth Intensive Care 2021 49 2 133 139 10.1177/0310057X20982623 33832336

[7] Iftikhar MH Raziq FI Laird-Fick H Uvular necrosis as a cause of throat discomfort after endotracheal intubation BMJ Case Rep 2019 12 7 e231227 10.1136/bcr-2019-231227 PMC6663153 31345834

[8] Goldin M Ji L Uvula necrosis, an atypical presentation of sore throat J Emerg Med 2013 44 1 185 186 10.1016/j.jemermed.2011.09.001 22494597

[9] Lee HJ Lim SY Pyon J Reconstruction of soft palate necrosis after endotracheal intubation J Craniofac Surg 2014 25 2 e176 e180 10.1097/SCS.0000000000000530 24621764

