# Supplementary figures and images for: Case Report of Post-Operative Uvular Necrosis Following Intubation
Source: J Educ Teach Emerg Med. 2025 Jul 31;10(3):V13–5. doi: 10.21980/J8065J (PMC12320993; doi:10.21980/J8065J)

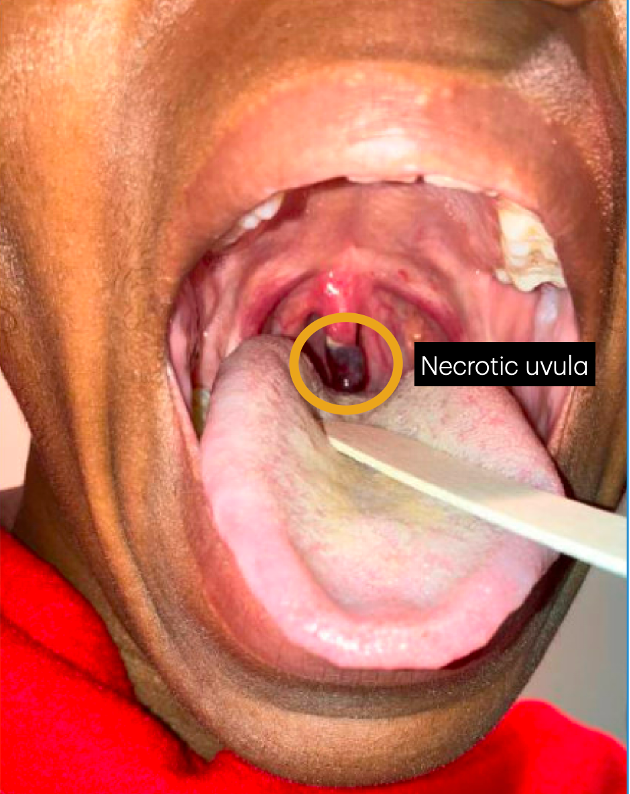

Supplement: Supplementary file 1 [file 10-3-V13-Supp1.jpg]

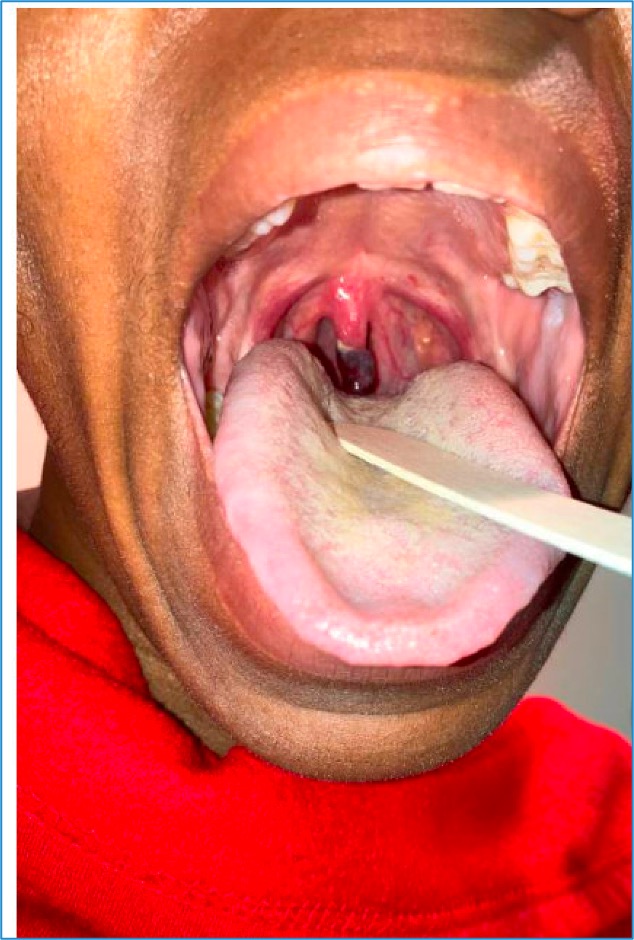

Supplement: Supplementary file 2 [file 10-3-V13-Supp2.jpg]
